# High Expression of C1ORF112 Predicts a Poor Outcome: A Potential Target for the Treatment of Low-Grade Gliomas

**DOI:** 10.3389/fgene.2021.710944

**Published:** 2021-11-22

**Authors:** Zhe Zhang, Zilong Tan, Qiaoli Lv, Lichong Wang, Kai Yu, Huan Yang, Huaizhen Liang, Tianzhu Lu, Yulong Ji, Junjun Chen, Wei He, Zhen Chen, Shuhui Chen, Xiaoli Shen

**Affiliations:** ^1^ Department of Neurosurgery, The Second Affifiliated Hospital of Nanchang University, Nanchang, China; ^2^ Jiangxi Key Laboratory of Translational Cancer Research, Jiangxi Cancer Hospital, Nanchang, China; ^3^ Department of Orthopaedics, Union Hospital, Tongji Medical College, Huazhong University of Science and Technology, Wuhan, China; ^4^ Department of Radiation Oncology, Jiangxi Cancer Hospital of Nanchang University, Nanchang, China

**Keywords:** C1ORF112, biomarker, immunoinfiltration, low-grade glioma, prognosis

## Abstract

**Background:** Glioma is the most common primary tumor of the central nervous system and is associated with poor overall survival, creating an urgent need to identify survival-associated biomarkers. C1ORF112, an alpha-helical protein, is overexpressed in some cancers; however, its prognostic role has not yet been explored in gliomas. Thus, in this study, we attempted to address this by determining the prognostic value and potential function of C1ORF112 in low-grade gliomas (LGGs).

**Methods:** The expression of C1ORF112 in normal and tumor tissues was analyzed using data from The Cancer Genome Atlas (TCGA), Chinese Glioma Genome Atlas (CGGA), Oncomine, and Rembrandt databases. The genetic changes of C1ORF112 in LGG were analyzed using cBioPortal. Survival analysis was used to evaluate the relationship between C1ORF112 expression and survival in patients with LGG. Correlation between immune infiltration and C1ORF112 expression was determined using Timer software. Additionally, data from three online platforms were integrated to identify the co-expressed genes of C1ORF112. The potential biological functions of C1ORF112 were investigated by enrichment analysis.

**Results:** C1ORF112 mRNA was highly expressed in LGGs (*p* < 0.01). Area under the ROC curve (AUC) showed that the expression of C1ORF112 in LGG was 0.673 (95% confidence interval [CI] = 0.618–0.728). Kaplan-Meier survival analysis showed that patients with high C1ORF112 expression had lower OS than patients with low C1ORF112 expression (*p* < 0.05). Multivariate analysis showed that high expression of C1ORF112 was an independent prognostic factor for the overall survival in patients from TCGA and CGGA databases. C1ORF112 expression was positively correlated with six immunoinfiltrating cells (all *p* < 0.001). The enrichment analysis suggested the enrichment of C1ORF112 and co-expressed genes in cell cycle and DNA replication.

**Conclusion:** This study suggested that C1ORF112 may be a prognostic biomarker and a potential immunotherapeutic target for LGG.

## Introduction

Glioma, the most commonly diagnosed and fatal type of primary tumor of the central nervous system (CNS) (Jiang et al., 2016), is often associated with poor prognosis (Demuth and Berens, 2004). As per the new classification of tumors of the CNS by the World Health Organization (WHO) in 2016, gliomas of the brain can be classified into four grades (I–IV) (Diamandis and Aldape, 2018; Aiman and Rayi, 2021) accordingly, gliomas are considered as “high-grade” and “low-grade”, wherein, high-grade gliomas show a high proliferative activity and strong invasion ability; whereas, low-grade gliomas (LGGs) show slow proliferation and relatively long survival time. Therefore, it is imperative to further investigate the key drivers of survival in LGGs and identify potential therapeutic targets to improve the overall prognosis.

The malignant development of a tumor is closely associated with gene expression. In 2012, Van Dam et al. determined that the mouse BC055324 gene (human homologous gene is C1ORF112) showed strong co-expression with cancer-related genes, such as, RAD51 and CCDC6 (van Dam et al., 2012). C1ORF112, an *α*-helical protein, is co-expressed with many genes in the BRCA-Fanconi anemia-associated DNA damage response pathway, including BRCA1, BRCA2, FANCD2, and FANCI (Nalepa and Clapp, 2018), and is also modified in some tumors with TP53 mutation (Edogbanya et al., 2021). Although, at present, only a few studies have reported the possible dysregulation of C1ORF112 in gastric cancer (Chen et al., 2020), this does suggest its biological and clinical significance in cancer. However, the underlying molecular functions of C1ORF112 and its expression and prognostic value in glioma remain undetermined.

Thus, in this study, we used gene expression and clinical data from Oncomine, The Cancer Genome Atlas (TCGA), and Chinese Glioma Genome Atlas (CGGA) to investigate the relationship between C1ORF112 and LGG, and determine its potential prognostic value in patients with LGG. Additionally, the genes co-expressed with C1ORF112 were collected, and their expression levels were verified in LGG. The results showed that C1ORF112 was significantly overexpressed in LGG samples and was an independent prognostic factor of the overall survival (OS) of patients with LGG. Further, C1ORF112 expression was closely related to the immune response of LGG, and played a crucial role in the malignant progression as well. Thus, C1ORF112, as a new prognostic factor, may be a new therapeutic target for the diagnosis and treatment of LGG.

## Materials and Methods

### Collection of Patient Data

We used TCGA database (TCGA-GBM; TCGA-LGG; https://portal.gdc.cancer.gov/) to download RNA-sequencing transcriptomic data and corresponding clinical information of 511 patients with LGG and 95 normal participants, 163 GBM patients and 207 normal participants. The inclusion criteria were defined as WHO II or III classified patients with complete prognostic information. From 161 patients with LGG and 28 normal participants, GlioVis (http://gliovis.bioinfo.cnio.es/) was used to download RNA-sequencing and corresponding clinical data, and used to verify C1ORF112 expression and prognostic potential in LGGs. Accordingly, patients with LGGs were then categorized into high and low expression groups according to the median expression value of C1ORF112. Additionally, C1ORF112 expression and clinical data of 381 LGG patients were downloaded from the CGGA (http://www.cgga.org.cn/) (Liu et al., 2018) database to analyze the relationship between C1ORF112 and patient prognosis. The patients we studied included both children and adults.

### Oncomine Database

Oncomine (https://www.oncomine.org/resource/login.html) database presents integrated RNA and DNA sequence data from the Gene Expression Omnibus, TCGA, and published literature. Using this database, we determined C1ORF112 expression in different types of cancers by setting the following criteria: *p* < 0.01, |log2 fold change| > 1.5, gene level 10%, and data type “mRNA”.

### cBioPortal Database

cBioPortal for Cancer Genomics (http://cBioportal.org) integrates data from more than 100 tumor genomic studies, and records the mutation site and possibility of a copy number variation at the mutation site. Here, we used high-throughput cBioPortal data to analyze the genetic changes associated with C1ORF112 in LGG samples.

### Immunoinfiltration Analysis

The relationship between C1ORF112 expression and immune cell infiltration in LGG samples from TCGA database was investigated using the Timer online website tool (Li et al., 2016).

### Enrichment and Protein-Protein Interaction Analyses

Multi Experiment Matrix (https://biit.cs.ut.ee/mem/index.cgi) (Kolde et al., 2012) and COXPRESdb (https://coxpresdb.jp/) (Obayashi et al., 2019) platforms were used to obtain C1ORF112 co-expression genes. Inclusion criterion was *p* < 0.05. According to TCGA data, genes with similar expression patterns as that of C1ORF112 in LGG were analyzed, and the inclusion criterion was *p* < 0.05. Using Database for Annotation, Visualization, and Integrated Discovery (DAVID; https://david.ncifcrf.gov/) (Huang et al., 2009), we performed gene ontology (GO) and Kyoto Gene and Genome Encyclopedia (KEGG) pathway analyses. A protein-protein interaction (PPI) network of C1ORF112 and co-expressed genes was constructed using STRING database (https://string-db.org/) (Szklarczyk et al., 2019), and co-expressed hub genes of C1ORF112 were obtained.

### Gene Expression Profiling Interactive Analysis Database

The Gene Expression Profiling Interactive Analysis (GEPIA) database (http://gepia.cancer-pku.cn/) (Tang et al., 2019) integrates TCGA data with GTEx normal tissue data to provide key interactive analysis and customization capabilities. Here, we used GEPIA to evaluate the expression and prognostic value of C1ORF112 in GBM, and to evaluate the expression and prognostic value of key co-expressed genes in LGG. The relationship between C1ORF112 and key co-expressed genes with OS was further analyzed.

### Statistical Analysis

Unpaired *t*-test was used to compare the expression levels of C1ORF112 between different groups, and *p* < 0.05 was considered significant. Receiver operating characteristic (ROC) curves were generated to evaluate the diagnostic performance of C1ORF112 expression using the SPSS. The median expression level of C1ORF112 was used to distinguish between the OS of patients with LGG. Kaplan-Meier method was used to plot the survival curves, and the OS differences between the groups were evaluated using log-rank test; here as well, *p* < 0.05 was considered significant. Univariate and multivariate analyses were performed to determine whether C1ORF112 expression was an independent prognostic marker in patients with LGG using the Cox proportional risk model. All statistical analyses were performed using R (version 4.0.2) and SPSS (version 26.0).

## Results

### C1ORF112 was Highly Expressed in Low-Grade Gliomas

**FIGURE 1:**
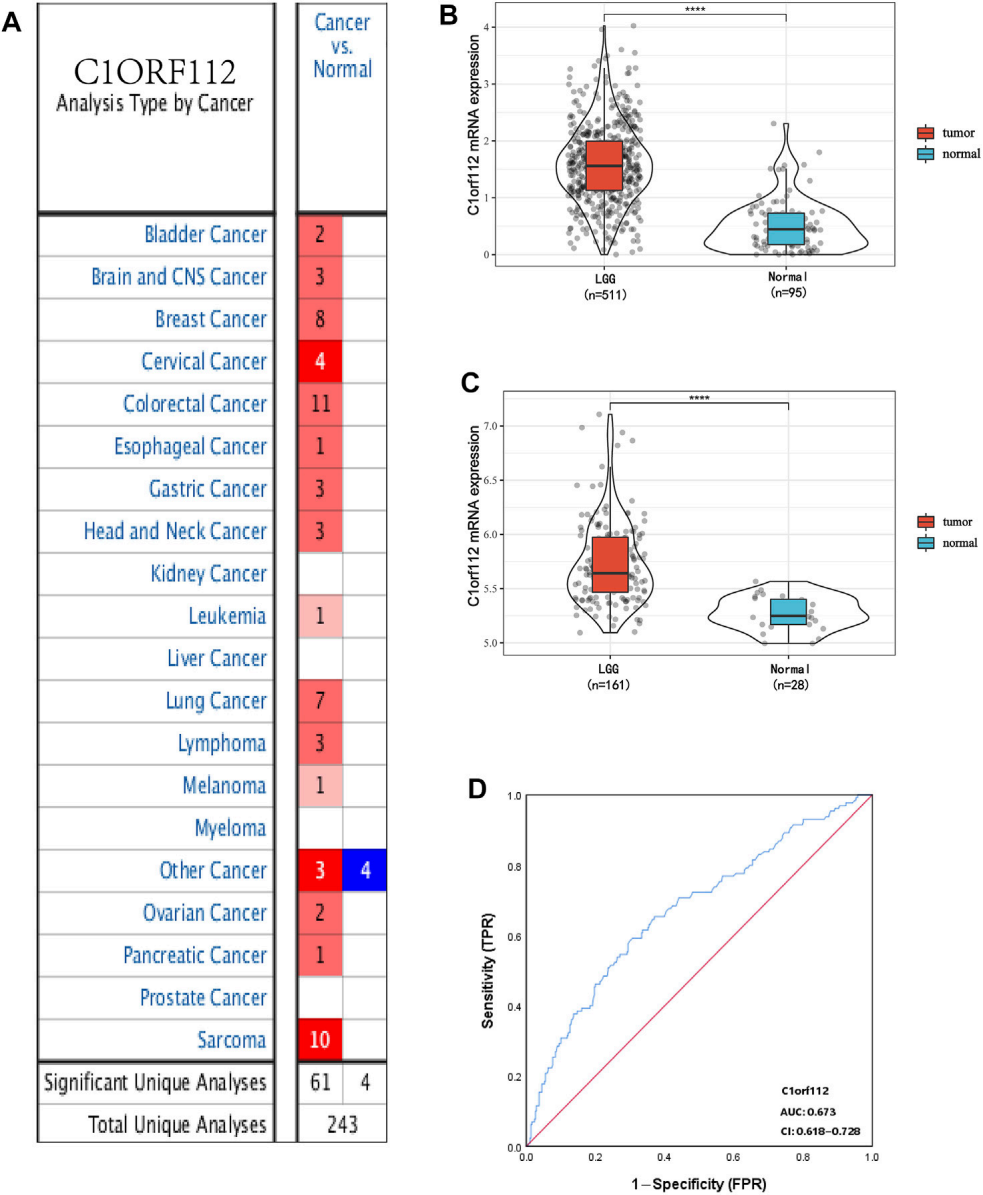
C1ORF112 expression between cancer and normal tissues in LGG patients. **(A)**. Transcriptional expression of C1ORF112 in different types of cancer diseases. C1ORF112 mRNA is highly expressed in low-grade glioma tissues in TCGA dataset **(B)** and Rembrandt dataset **(C)**. **(D)** Receiver operating characteristic analysis (ROC) of C1ORF112 in LGG. ****p < 0 .0001.

Using data from Oncomine, we analyzed the transcriptional levels of C1ORF112 in different cancer types. Compared with the normal tissues (*p* < 0.01, |log2 fold change| >1.5), we found that C1ORF112 was upregulated in almost all cancer types (Figure 1A), including colorectal cancer, breast cancer, lung cancer, sarcomas, and tumors of the CNS. Further, multiple data sets showed that C1ORF112 expression was significantly elevated in the CNS (Table 1). For instance, in the Sun Brain dataset, C1ORF112 expression in diffuse astrocytomas was 2.313 times higher than that in normal tissues (*p* = 2.23E-4). Similarly, in the French Brain dataset, C1ORF112 expression was 1.9 times higher (*p* = 0.001) in anaplastic oligodendrocytomas and 1.544 times higher (*p* = 1.13E-5) in anaplastic oligodendrocytomas than in normal tissues. We have analyzed the relationship between C1orf112 and high-grade glioma using TCGA database. The results indicated that C1orf112 was highly expressed in high-grade gliomas, but its prognostic value was not statistically significant (*p* = 0.59) (Supplementary Figures S1A,B). We then obtained C1ORF112 expression profiling data of 511 patients with LGG using TCGA, and observed that C1ORF112 was significantly upregulated in the tumor tissues than in the non-tumor tissues (Figure 1B; *p* < 0.01). In addition, we performed a validation using C1ORF112 profiling data from the Rembrandt database (Figure 1C; *p* < 0.01), and observed that C1ORF112 was highly expressed in LGGs. Further, area under the ROC curve (AUC) showed that the expression of C1ORF112 in LGG was 0.673 (95% confidence interval [CI] = 0.618–0.728; Figure 1D).

**TABLE 1:** Significant changes of C1ORF112 expression in transcription level between Brain glioma and Normal brain tissues (ONCOMINE).

	Types of brain glioma VS normal	Fold change	*p* Value	*t*-Test	Ref
C1ORF112	Diffuse Astrocytoma	2.313	2.23E-4	4.004	Sun Brain
	Anaplastic Oligoastrocytoma	1.900	0.001	5.828	French Brain
	Anaplastic Oligodendroglioma	1.544	1.13E-5	5.545	French Brain

### Correlation of C1ORF112 With Clinical Features in Low-Grade Gliomas

**FIGURE 2:**
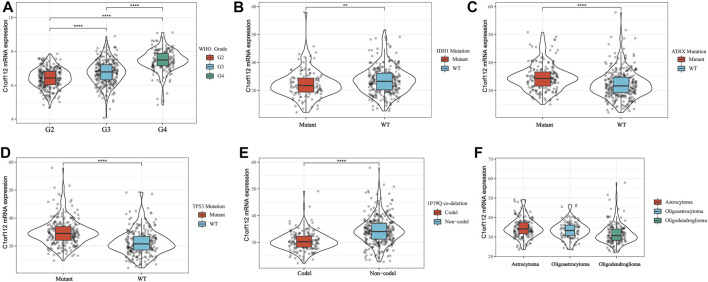
C1ORF112 mRNA was related to WHO Grade **(A)**, IDH1 **(B)**, ATRX **(C)**, TP53 **(D)**, status of 1P19Q co-deletion **(E)**, and LGG subtypes **(F)**. **p < 0.01, ****p < 0 .0001.

**FIGURE 3:**
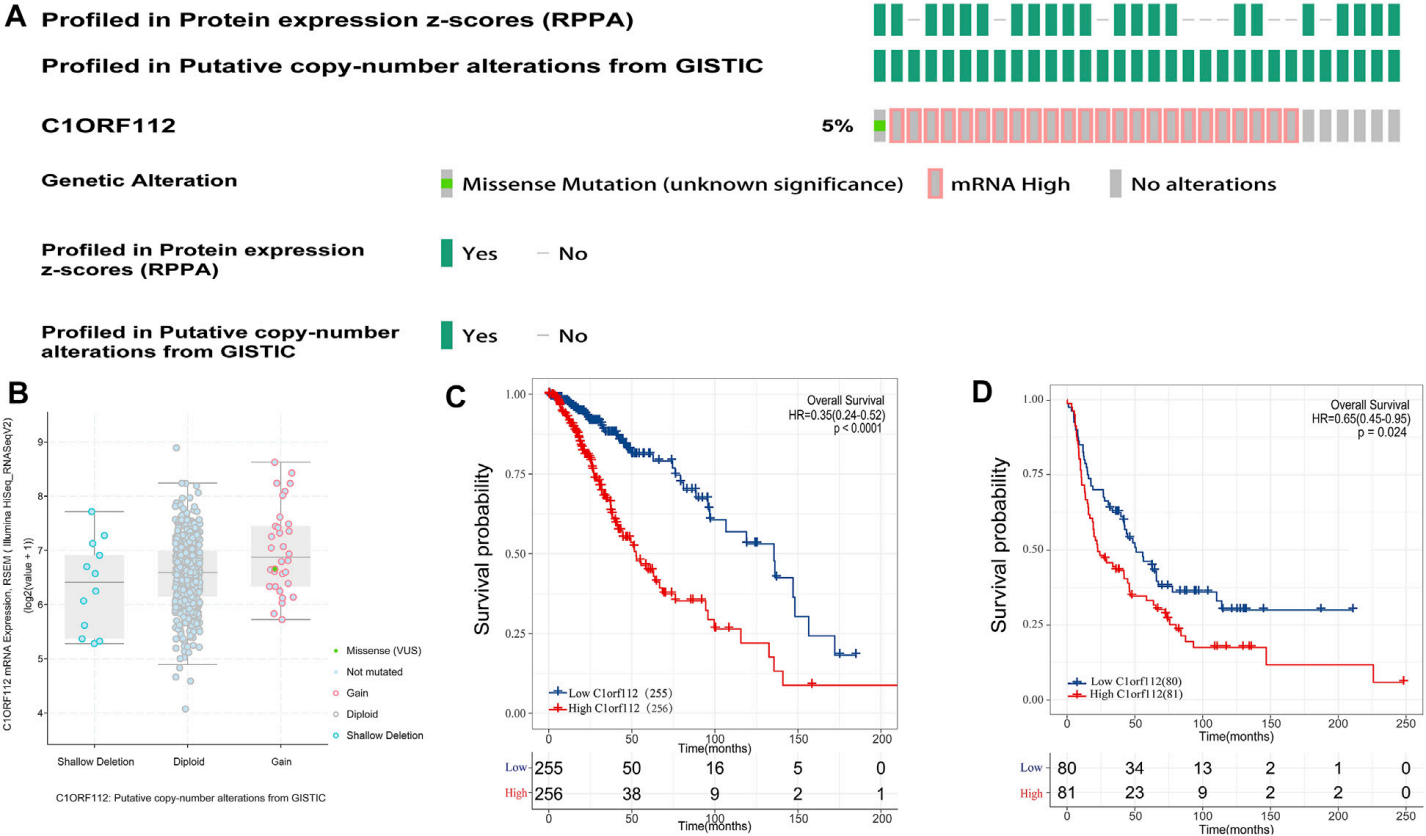
Genetic alterations and prognostic value of C1ORF112 expression in low-grade gliomas. **(A)** Mutation rate of C1ORF112 in LGGs. **(B)** Putative copy number alterations of C1ORF112 in LGGs. **(C)** Survival curves of OS from TCGA dataset (*n* = 511). **(D)** Survival curves of OS from Rembrandt dataset (*n* = 161).

Using cBioPortal, we found that in LGGs, C1ORF112 had a mutation with a relatively low rate of genetic change. Therefore, the role of highly expressed C1ORF112 in LGG development may not be mediated by mutations or amplification (Figures 3A,B). We analyzed the relationship between C1ORF112 and WHO grades using TCGA database, and found that the C1ORF112 mRNA expression was positively correlated with the WHO grades (Figure 2A). In addition, We analyzed the relationship between C1ORF112 expression and mutational status of IDH1, ATRX and 1P19Q co-deletion status (Figures 2B–E). The relationship between LGG subtype (includes Astrocytoma, Oligoastrocytoma and Oligodendrogliom) and C1ORF112 expression was also analyzed (Figure 2F).

### Correlation Between C1ORF112 Overexpression and Overall Survival of Low-Grade Gliomas

To investigate the relationship between C1ORF112 expression and OS, we classified 255 patients into the high expression group and 256 patients into the low expression group according to the median expression value of C1ORF112 in PANCAN-LGG in TCGA. Kaplan-Meier survival analysis showed that patients with high C1ORF112 expression had lower OS than patients with low C1ORF112 expression (*p* < 0.001; Figure 3C). To further validate the prognostic value of C1ORF112 expression in LGG, data from 161 patients in the Rembrandt database were analyzed (Figure 3D; *p* < 0.05). The results showed that patients with high expression of C1ORF112 in LGG had significantly lower OS than those with low expression of C1ORF112. Furthermore, univariate analysis using TCGA data showed that C1ORF112, age, and grade were high-risk factors (Table 2). Multivariate analysis confirmed that C1ORF112 was an independent prognostic factor for the OS of LGG [hazard ratio (HR) = 1.554, 95% CI = 1.040–2.321; *p* = 0.031; Table 2]. Similarly, validation using the CGGA database confirmed that C1ORF112 was indeed an independent prognostic factor for the OS of LGG (HR = 1.500, 95% CI = 1.109–2.209, *p* = 0.009; Table 2).

**TABLE 2:** Univariate and multivariate analysis of C1ORF112 expression profile in TCGA database and CGGA database.

Datasets	Univariate			Multivariate	
	Variable	HR (95% CI)	P	HR (95% CI)	P
TCGA	Age	4.764 (3.042–7.462)	<0.001	4.998 (3.111–8.030)	<0.001
	Gender	1.159 (0.796–1.686)	0.441	1.053 (0.715–1.550)	0.795
	Histological_type	0.752 (0.607–0.932)	0.009	0.800 (0.634–1.010)	0.061
	Histologic_grade	3.630 (2.397–5.496)	<0.001	3.067 (2.015–4.668)	<0.001
	Idh1_mutation	0.895 (0.544–1.472)	0.663	0.957 (0.572–1.601)	0.867
	C1ORF112	1.876 (1.284–2.740)	0.001	1.554 (1.040–2.321)	0.031
CGGA	Age	1.486 (0.760–2.905)	0.246	1.012 (0.507–2.023)	0.972
	Gender	1.085 (0.811–1.452)	0.583	1.146 (0.846–1.553)	0.378
	Histological_type	1.224 (1.114–1.343)	<0.001	0.558 (0.395–0.786)	0.001
	Histologic_grade	2.932 (2.096–4.100)	<0.001	17.005 (5.715–50.604)	<0.001
	Idh1_mutation	0.463 (0.339–0.630)	<0.001	0.517 (0.367–0.728)	<0.001
	C1ORF112	1.772 (1.324–2.372)	<0.001	1.500 (1.109–2.209)	0.009

### Relationship Between C1ORF112 and Immune Infiltration

**FIGURE 4:**
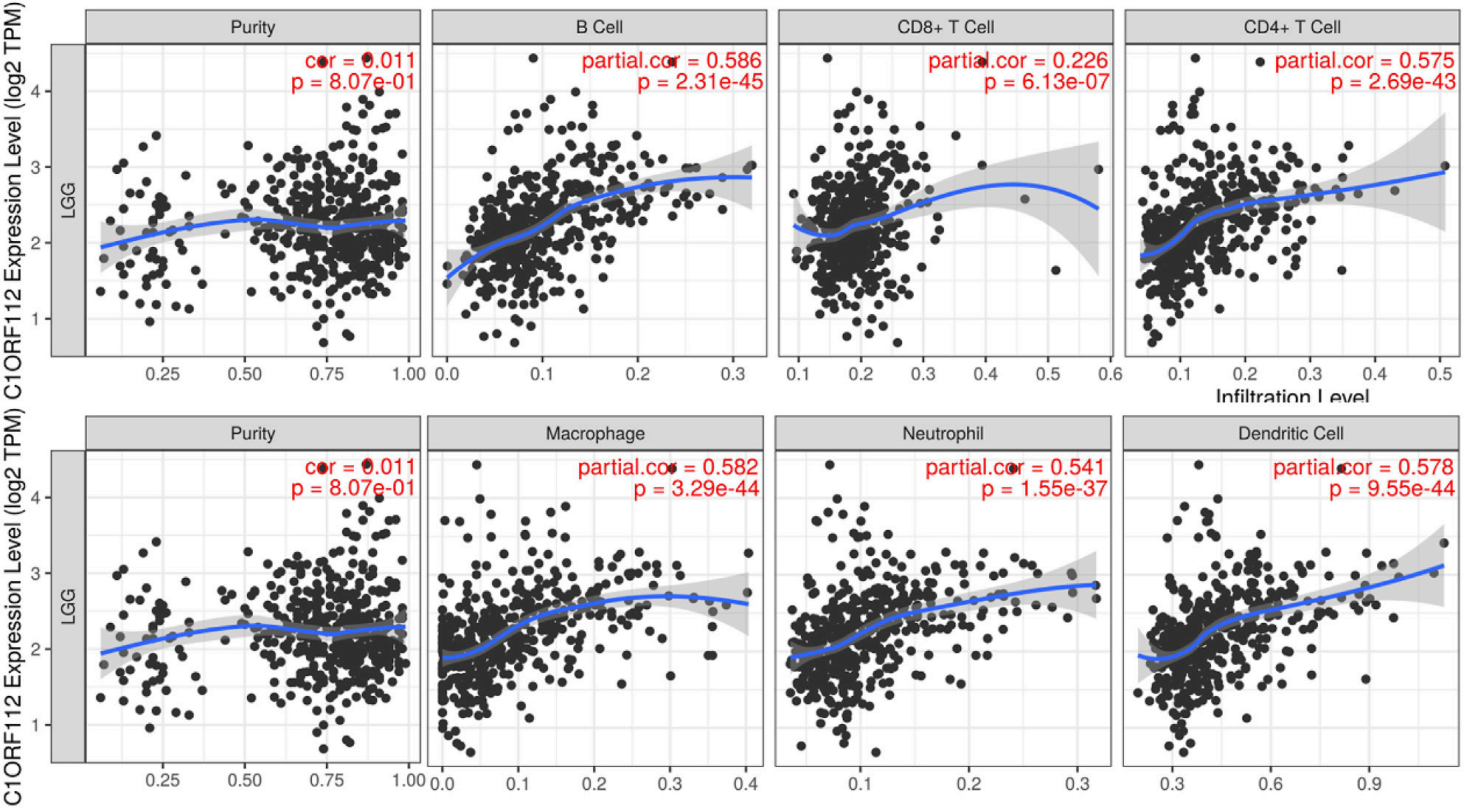
The relationship between C1ORF112 and the level of immune infiltration.

Immune cell infiltration may be an important pathophysiological factor in the development of glioma. We analyzed the relationship between C1ORF112 expression and infiltration of six common immune cells: B cells, CD8^+^ T cells, CD4^+^ T cells, macrophages, neutrophils, and dendritic cells. C1ORF112 expression was positively correlated with all six immune cells (*p* < 0.001; Figure 4), indicating that patients with high C1ORF112 expression in LGGs had higher immune cell infiltration than patients with low C1ORF112 expression.

### Enrichment and Protein-Protein Interaction Analyses of C1ORF112 Co-expressed Genes

**FIGURE 5:**
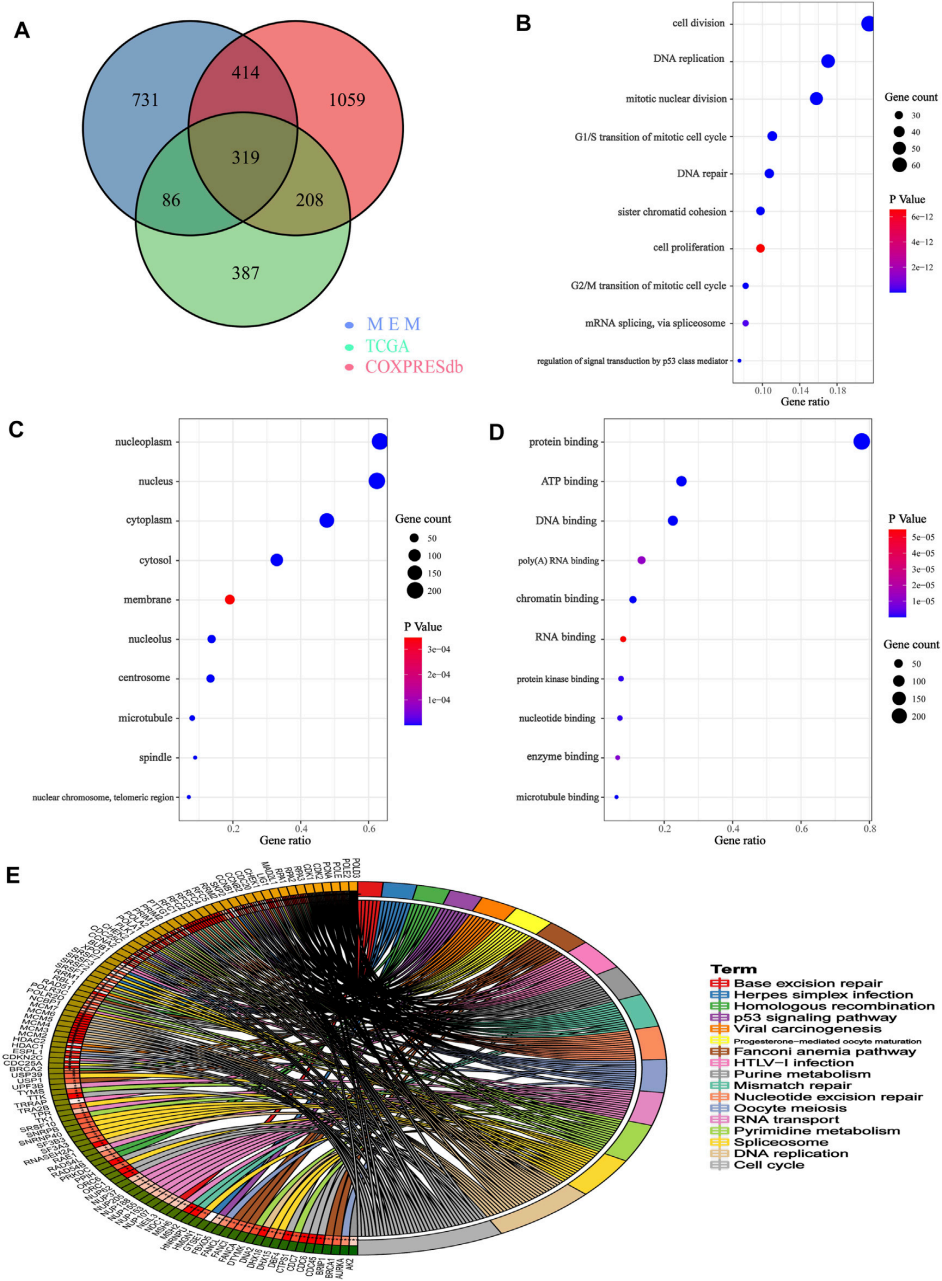
Functional enrichment of C1ORF112 and its co-expressed genes in low-grade gliomas. **(A)** Venn diagram of C1ORF112 co-expressed genes in LGG. **(B)** Enriched GO terms in the “biological process” category. **(C)** Enriched GO terms in the “cellular component” category. **(D)** Enriched GO terms in the “molecular function” category. **(E)** Kyoto Encyclopedia of Genes and Genomes Pathway.

**FIGURE 6:**
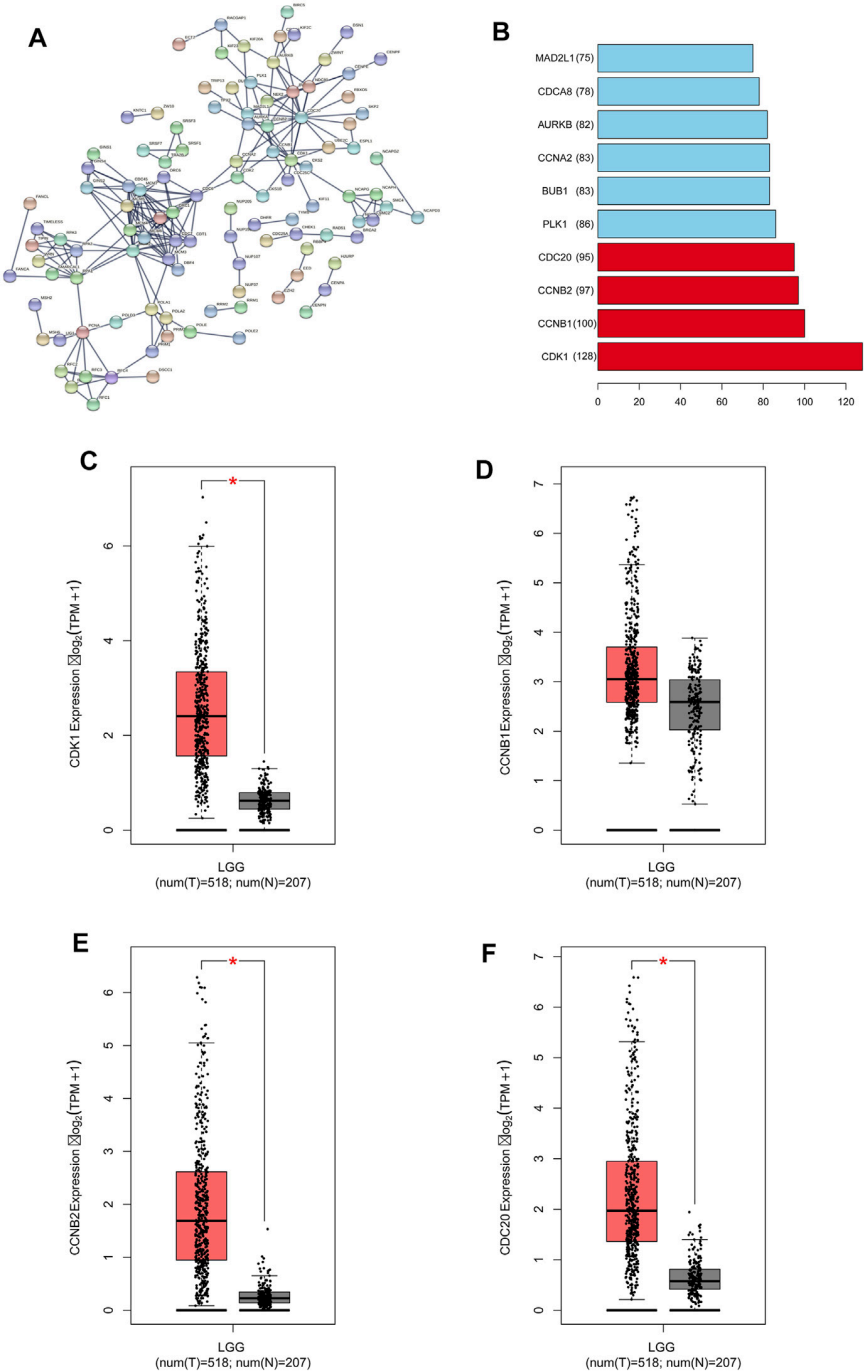
C1ORF112 and its co-expressed genes in a protein-protein interaction network in low-grade gliomas. **(A)** PPI network. **(B)** Top 10 of key co-expressed genes of C1ORF112. The expression level of key co-expressed genes in LGGs was : **(C)** CDK1; **(D)** CCNB1; **(E)** CCNB2; **(F)** CDC20. *p < 0 .05.

We obtained 2000 C1ORF112 co-expressed genes from Multi Experiment Matrix and Coxpresdb platforms. Then, 1,000 genes similar to C1ORF112 expression patterns in LGGs were obtained by calculating the TCGA database. Finally, 319 overlapping genes (that were overlapping in all three databases) were considered as the co-expressed genes of C1ORF112 in LGGs (Figure 5A). GO enrichment analysis showed that C1ORF112 and the co-expressed genes were mainly enriched in cell division, DNA repair, and ATP binding (Figures 5B–D). KEGG analysis revealed that C1ORF112 and the co-expressed genes were mainly enriched in cell cycle, DNA replication, pyrimidine metabolism, and RNA transport (Figure 5E). PPI analysis showed that CDK1, CCNB1, CCNB2, CDC20 were the key co-expressed genes of C1ORF112 in LGGs (Figures 6A,B).

### Correlation Between the Key C1ORF112 Co-expressed Genes and Low-Grade Gliomas

**FIGURE 7:**
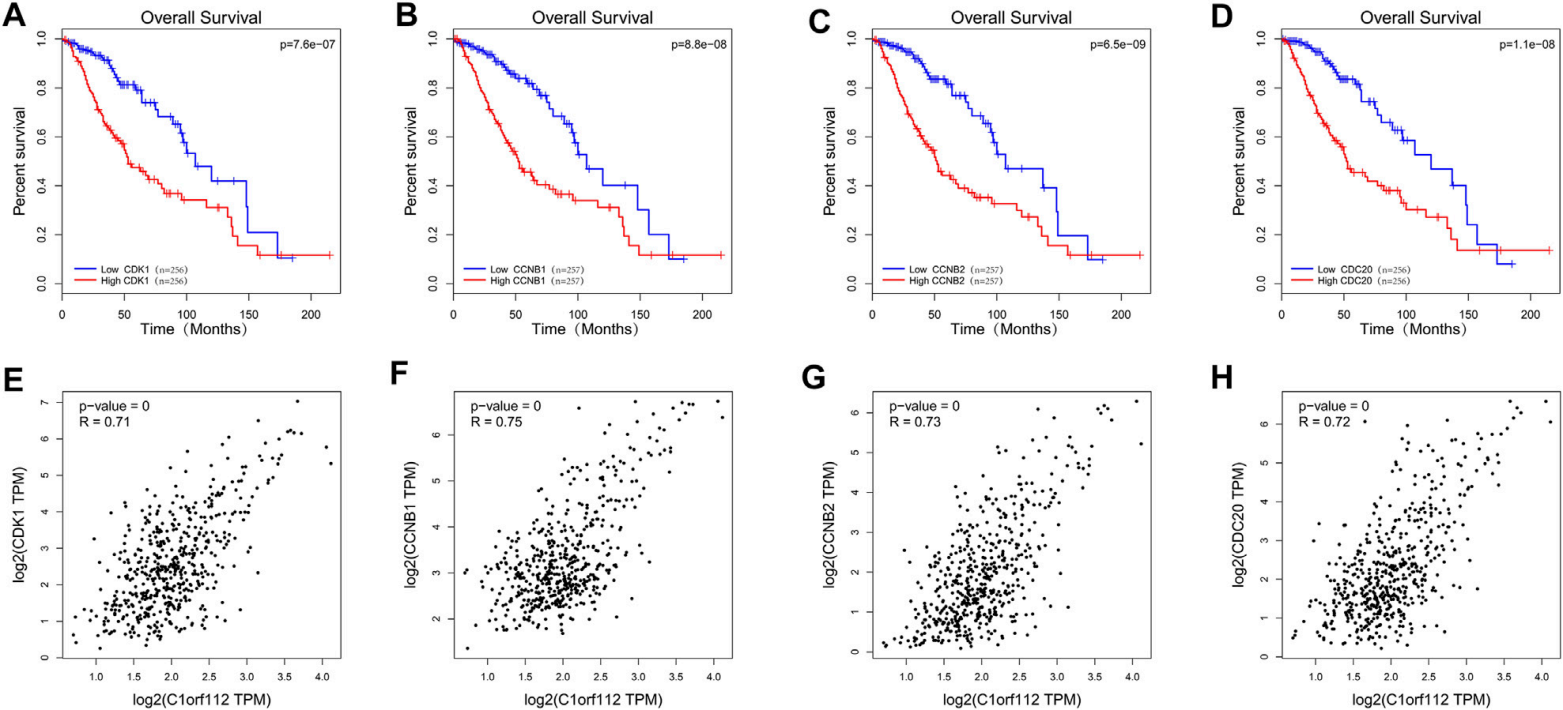
The TCGA database was used to analyze the prognostic value and correlation of co-expressed genes. Survival curves of OS: **(A)** CDK1; **(B)** CCNB1; **(C)** CCNB2; **(D)** CDC20. Correlation between C1ORF112 and key co-expressed genes: **(E)** CDK1; **(F)** CCNB1; **(G)** CCNB2; **(H)** CDC20.

We used GEPIA to analyze the RNA-sequencing data of 518 LGG tissues from TCGA and 207 normal samples from the GTEx project, and found that CDK1, CCNB1, CCNB2, and CCDC20 were highly expressed in the LGG tissues and poorly expressed in the normal tissues (Figures 6C–F). Kaplan-Meier survival analysis showed that LGG patients with high CDK1, CCNB1, CCNB2, and CDC20 expression had a significantly lower OS than patients with low CDK1 expression (*p* < 0.001; Figures 7A–D). LGG analysis in TCGA showed that C1ORF112 was significantly positively correlated with the key co-expressed genes (Figures 7E–H).

## Discussion

Glioma, a highly heterogenous tumor, is the most commonly diagnosed tumor in the CNS and is associated with poor OS (Ostrom et al., 2018; Waker and Lober, 2019). While there have been a few studies on C1ORF112 mRNA, to our knowledge, no study has investigated the correlation between C1ORF112 and LGG. In this study, 163 GBM tissues and 207 normal tissues were studied in TCGA database. Our results showed that C1ORF112 was significantly overexpressed in GBM, but the prognostic analysis was not statistically significant. This might be explained by the small number of glioblastoma samples and different molecular mechanisms between LGG and GBM. Therefore, we integrated and screened clinical RNA-sequencing data from TCGA, CGGA, and Rembrandt databases, and obtained a total of 1053 LGG tissues and 123 normal tissues. Our results revealed that C1ORF112 was significantly overexpressed in LGGs with ATRX mutation, TP53 mutation, 1p19q non-codel, or Astroglioma. In addition, univariate and multivariate analyses showed that C1ORF112 expression was an independent prognostic factor for LGG. The enrichment analysis showed that C1ORF112 and its co-expressed genes were associated with cell cycle, DNA replication, pyrimidine metabolism, nucleotide excision, and repair, RNA transport, purine metabolism, and the Fanconi anemia pathway. Therefore, C1ORF112 may play an important role in glioma pathogenesis, and may be a potential LGG biomarker.

High C1ORF112 mRNA expression has been previously reported in breast cancer (Leo et al., 2005), gastric cancer (Chen et al., 2020), desmoid tumors (Bowden et al., 2007), bladder cancer (Sanchez-Carbayo et al., 2007), head and neck squamous cell carcinoma (Renkonen et al., 2017), cervical cancer, and others. Increased copy number of C1ORF112 has been reported in breast cancer studies (Gonzalez-Perez et al., 2013; Rubio-Perez et al., 2015). However, to date, the expression of C1ORF112 in LGG has not been studied, and the expression of C1ORF112 in other cancers has only been verified *via* the co-expression analysis of related genes. In this study, we confirmed that C1ORF112 was significantly overexpressed in most tumors in TCGA database. Further analysis showed that C1ORF112 was highly expressed in LGG than in normal tissues (*p* < 0.001). Additionally, the AUC was 0.673. Together, these results suggest that C1ORF112 has the potential to be a diagnostic marker for many cancers, including LGG. The expression of C1ORF112 is closely related to the survival of patients with endometrial cancer, wherein, higher the expression, worse the prognosis. Notably, our study is the first to show that C1ORF112 expression may influence the prognosis in LGG. By analyzing TCGA-LGG data, we found that patients with high C1ORF112 mRNA expression had poor OS, and this was an independent prognostic factor for OS and progression-free survival. This result was supported by clinical LGG data from the Rembrandt database as well. In addition, multivariate analysis showed that C1ORF112 was an independent prognostic factor for LGG. Therefore, it is necessary to further investigate the role of C1ORF112 in LGG.

To explore the possible mechanism of C1ORF112 in LGG, we performed enrichment analysis of C1ORF112 and its co-expressed genes, and found that they were enriched in cell cycle, DNA replication, Fanconi anemia, Mismatch repair, Nucleotide excision repair etc. Previous studies have found that C1ORF112 may influence the Fanconi anemia pathway or its regulation (Liu et al., 1998; Auerbach, 2009). Zhaojing et al. has reported that LINC00152 promotes the proliferation, migration, and invasion of gastric cancer cells *in vitro* through the cell cycle pathway (Zhao et al., 2015). Qiuni et al. reported that cullin-7 is a predictor of poor prognosis in patients with breast cancer, and is involved in the regulation of breast cancer cells by regulating the cell cycle (Qiu et al., 2018). Yun et al. reported the involvement of the cell cycle pathway in cerebellar meningeal metastasis of non-small cell lung cancer (Fan et al., 2018). Hung-Wei et al. reported that overexpression of cell cycle regulating nuclear cell protein L2DTL was associated with the progression and poor prognosis of hepatocellular carcinoma (Pan et al., 2006). Thus, the cell cycle not only plays an important role in tumor regulation, but also affects the prognostic evaluation (Williams and Stoeber, 2012). C1ORF112 and its co-expressed genes were positively correlated with the cell cycle. Four key co-expression genes (CDK1, CCNB1, CCNB2, and CDC20) of C1ORF112 were further analyzed. CDK1-mediated perturbations in chromosome stability and G2/M control that promotes cell cycle progression are key tumorigenic events (Asghar et al., 2015). Overexpression of FOXM1 and upregulation of CCNB1 leads to a malignant phenotype (Katoh et al., 2013). CCNB2 is overexpressed in non-small cell lung cancer and is closely associated with poor prognosis (Qian et al., 2015). High expression of CDC20 is significantly associated with reduced survival of most human tumors (Wang et al., 2018). Therefore, we speculate that C1ORF112 may be involved in the progression of LGG *via* the cell cycle. DNA damage repair is a phenomenon that DNA molecules of normal cells are damaged followed by a series of activation of various enzymes to restore their structures (Ciccia and Elledge, 2010). The mechanism plays an important role in maintaining gene stability (Yan et al., 2016). DNA damage repair includes four types: nucleotide excision repair, base excision repair, recombination repair, and mismatch repair. DNA damage repair is quite important for regulating the therapeutic response of cancer (Squatrito and Holland, 2011). Numerous chemotherapeutic drugs exert anti-tumor effects through DNA damage (Pang et al., 2020). Temozolomide, for example, is the first-line drugs to kill glioma cells by damaging their DNA. Our analysis showed that C1ORF112 and its co-expressed genes were involved in nucleotide excision repair and mismatch repair. Therefore, we speculate that C1ORF112 may be involved in the progression of LGG through facilitating DNA damage repair. Finally, we concluded that four key co-expression genes of C1ORF112 (CDK1, CCNB1, CCNB2, and CDC20) were highly expressed in LGG, and the high expression of these genes was closely associated with poor prognosis in patients with LGG.

Infiltration of immune cells plays a crucial role in tumor growth, metastasis, and treatment response (Gieryng et al., 2017). Therefore, we analyzed the correlation between C1ORF112 and infiltrating immune cells. The results showed that the expression of C1ORF112 was negatively correlated with tumor purity and positively correlated with the infiltration of CD4^+^ T cells, CD8^+^ T cells, B cells, neutrophils, dendritic cells, and other immune cells. This is the first report of the potential involvement of C1ORF112 in immunity. According to the immune response, low-grade gliomas can form an immunosuppressive tumor microenvironment similar to other tumors that impair T cell antitumor responses *via* immune checkpoint inhibition pathways. Immune checkpoint inhibitors (i.e., monoclonal antibody inhibitors) were develped to block these inhibitory signaling pathways, activate systemic immunity, and thus enhance T cell activity. Typically, Programmed cell death 1 (PD-1) and its ligand Programmed cell death ligand 1 (PD-L1) mediates tumor immunosuppression by promoting T cell apoptosis and Treg induction (Nduom et al., 2016). Therefore, PD-1/PD-L1 is an important immunosuppressive interaction for tumor cells to escape from the immune killing of the matrix. Dung et al. reported the mismatch repair deficiency predicts response of tumors to PD-1 blockade (Le et al., 2017). Our current study found that C1ORF112 and its co-expressed genes are functionally enriched in mismatch repair. However, further studies are needed to evaluate the role C1ORF112 and mismatch repair on PD-L1 immune checkpoint therapy. Immunotherapy aims to strengthen the immune system of a patient to recognize and attack tumor cells. Therefore, C1ORF112 may be a target for future immunotherapy.

Through this study, we have improved the understanding of the relationship between C1ORF112 and LGG; however, there are still some limitations of our study. The knock-down and knock-out experiments of C1ORF112 is supposed to be performed to explore and verify its function in LGG in our future study. For example, proliferation, migration, invasion, and immune response of LGG cells, and *in vivo* study using LGG mouse model can be performed to verify the mechanism of C1ORF112. Translating these cell cycle-associated biomarkers into practical clinical applications also requires further investigation. In conclusion, the overexpression of C1ORF112 mRNA in LGG was closely related to the poor prognosis of patients with LGG. Enrichment analysis showed that C1ORF112 may regulate the progression of LGG *via* the cell cycle, affect the prognosis of patients with LGG, and thus play a potential role as a carcinogenic factor. Finally, this study suggests that C1ORF112 may be a potential biomarker for the diagnosis and prognosis of LGG, and a potential immunotherapeutic target.

## Data Availability

The datasets presented in this study can be found in online repositories. The names of the repository/repositories and accession number(s) can be found in the article/Supplementary Material.

## Supplementary Material

Supplementary Figure S1 C1ORF112 expression and prognostic value between cancer and normal tissues in GBM patients. **(A)** C1ORF112 mRNA is highly expressed in GBM tissues in TCGA dataset. **(B)** Survival curves of OS from TCGA dataset (*n*=162).

## Abbreviations

CGGA, Chinese Glioma Genome Atlas; CNS, central nervous system; DAVID, Database for Annotation, Visualization, and Integrated Discovery; GBM, Glioblastoma; GEPIA, Gene Expression Profiling Interactive Analysis; GO, gene ontology; KEGG, Kyoto Encyclopedia of Genes and Genomes; LGG, low-grade glioma; OS, overall survival; PPI, protein-protein interaction; ROC, receiver operating characteristic curve; TCGA, The Cancer Genome Atlas; WHO, World Health Organization.
