# Supplementary figures and images for: High Expression of C1ORF112 Predicts a Poor Outcome: A Potential Target for the Treatment of Low-Grade Gliomas
Source: Front Genet. 2021 Nov 22;12:710944. doi: 10.3389/fgene.2021.710944 (PMC8645850; doi:10.3389/fgene.2021.710944)

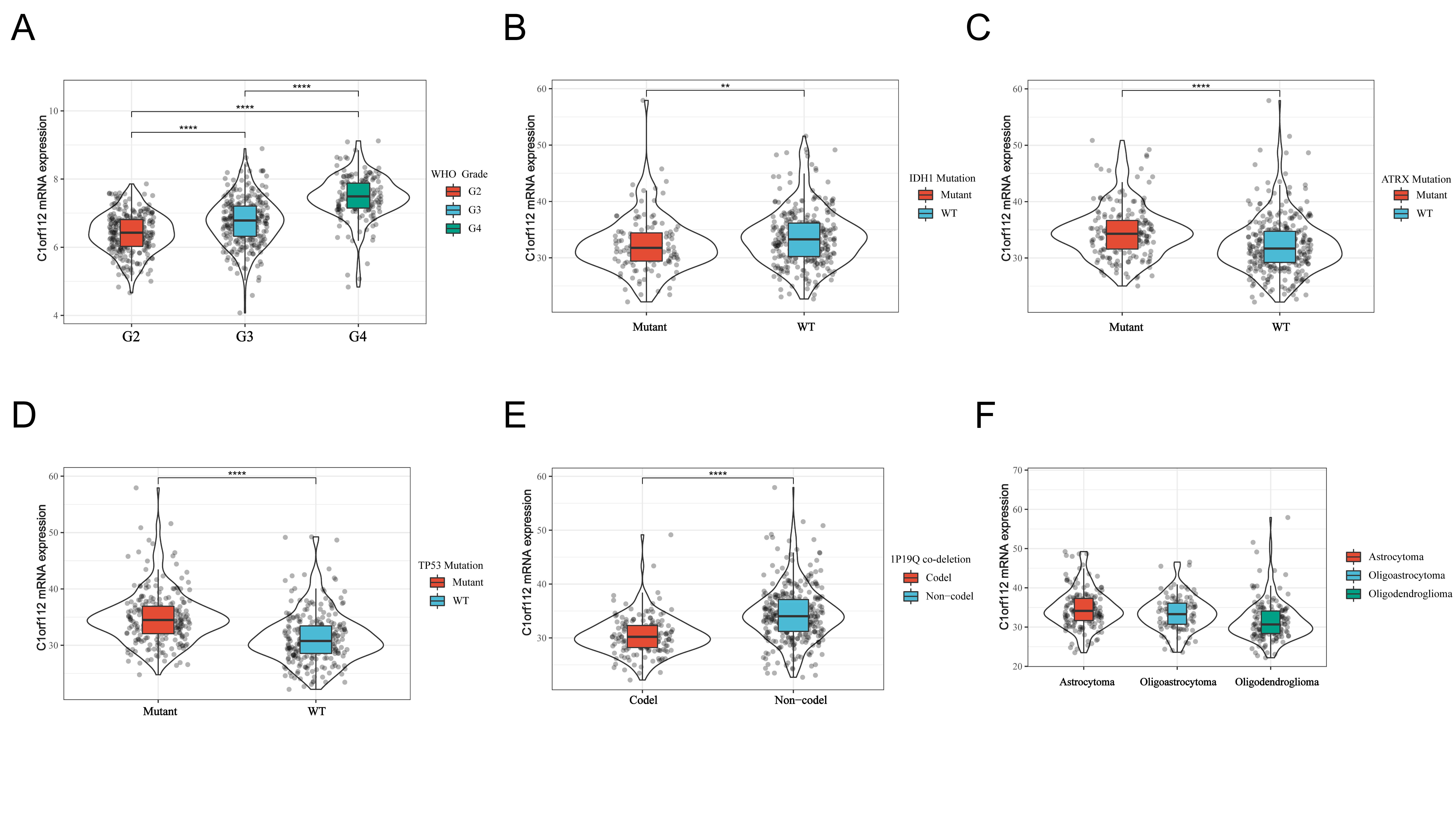

Supplement: Supplementary file 1 [file Image2.TIF]

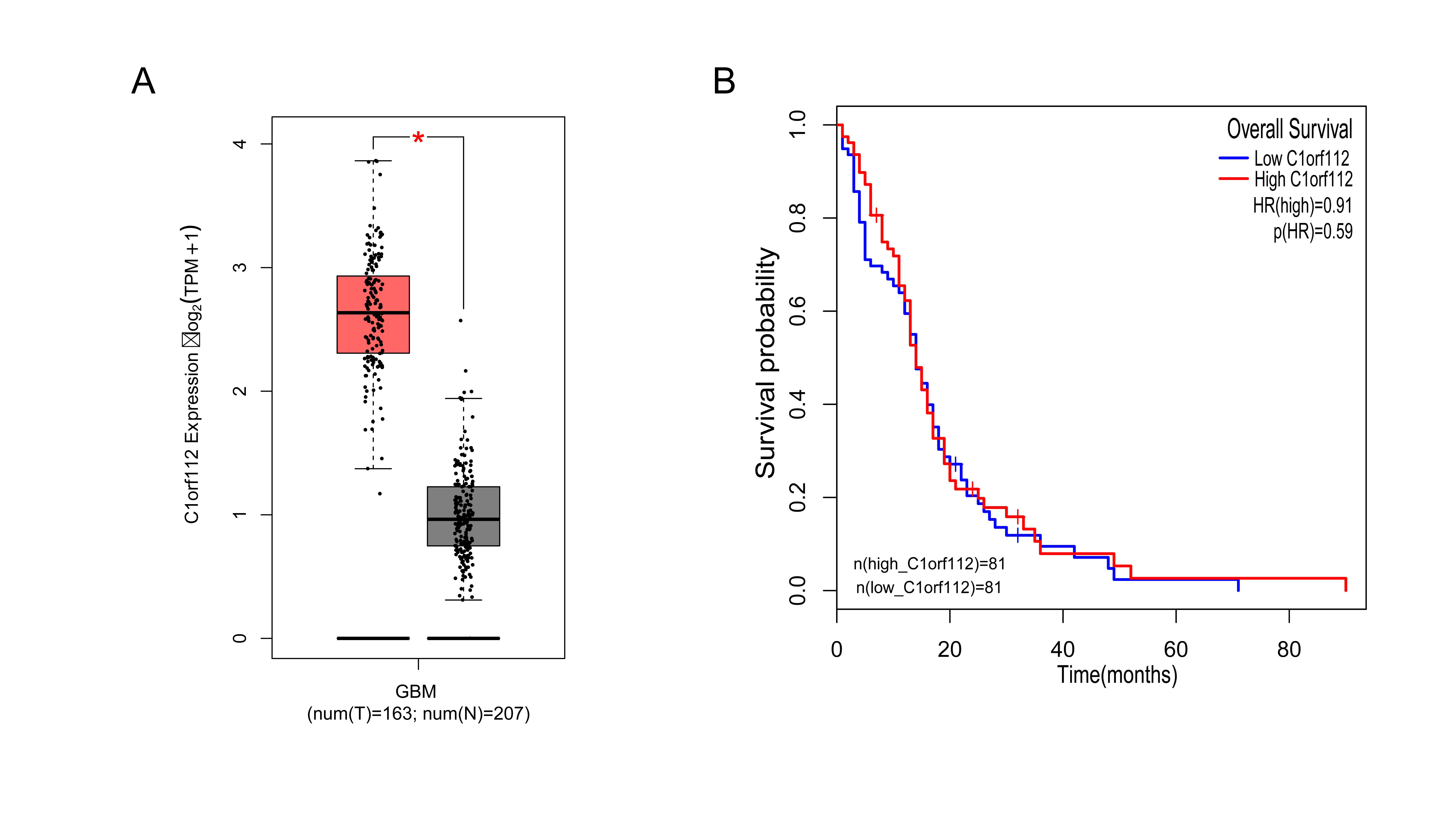

Supplement: Supplementary file 2 [file Image1.TIF]
